# A Ploidy-Sensitive Mechanism Regulates Aperture Formation on the Arabidopsis Pollen Surface and Guides Localization of the Aperture Factor INP1

**DOI:** 10.1371/journal.pgen.1006060

**Published:** 2016-05-13

**Authors:** Sarah H. Reeder, Byung Ha Lee, Ronald Fox, Anna A. Dobritsa

**Affiliations:** Department of Molecular Genetics and Center for Applied Plant Science, the Ohio State University, Columbus, Ohio, United States of America; Peking University, CHINA

## Abstract

Pollen presents a powerful model for studying mechanisms of precise formation and deposition of extracellular structures. Deposition of the pollen wall exine leads to the generation of species-specific patterns on pollen surface. In most species, exine does not develop uniformly across the pollen surface, resulting in the formation of apertures–openings in the exine that are species-specific in number, morphology and location. A long time ago, it was proposed that number and positions of apertures might be determined by the geometry of tetrads of microspores–the precursors of pollen grains arising via meiotic cytokinesis, and by the number of last-contact points between sister microspores. We have tested this model by characterizing Arabidopsis mutants with ectopic apertures and/or abnormal geometry of meiotic products. Here we demonstrate that contact points *per se* do not act as aperture number determinants and that a correct geometric conformation of a tetrad is neither necessary nor sufficient to generate a correct number of apertures. A mechanism sensitive to pollen ploidy, however, is very important for aperture number and positions and for guiding the aperture factor INP1 to future aperture sites. In the mutants with ectopic apertures, the number and positions of INP1 localization sites change depending on ploidy or ploidy-related cell size and not on INP1 levels, suggesting that sites for aperture formation are specified before INP1 is brought to them.

## Author Summary

Deposition of extracellular materials next to a cell can protect the cell, change its morphology, or help it to move and communicate. To be able to perform such functions, extracellular materials must be deposited very precisely, but how cells achieve such precision is mostly unknown. A beautiful example of a very precisely deposited extracellular structure is the wall surrounding pollen grains. Pollen from different species often looks remarkably different–in part, because wall materials are deposited at some places on pollen surface and absent from others. The places where the wall is absent are called apertures and they are species-specific in shape, number, and positions. This suggests that developing pollen consistently marks some of its surface regions as different from the rest of the surface to allow them to develop into apertures. Aperture number and positions were long thought to be determined by geometric arrangement of pollen precursors arising during meiotic division and by the number of last-contact points between these cells. Here we challenge this model by using Arabidopsis mutants with abnormal arrangement of meiotic products or the number of last-contact points and by demonstrating that these factors are neither necessary nor sufficient for aperture placement. In contrast, we find that a mechanism sensitive to pollen ploidy–with cells responding to factors dependent either on ploidy itself or on cell size–is very important for aperture number and positions.

## Introduction

Deposition of extracellular materials is used by cells to control cell morphology, growth, and motility, to promote tissue formation, and to protect themselves from harmful influences [1–3]. In the tissues and organisms in which cells are surrounded by an extracellular matrix or by cell walls, the regulated production and deposition of these extracellular structures at precisely the right places is often critical for organism development, survival, or propagation. In animals, the regulated placement of extracellular matrix contributes, among other processes, to axonal navigation [4], control of egg cell shape [5,6], tissue asymmetry generation [7], and wound healing [8]. In plants, similarly precise deposition or modification of cell walls is required for such important and diverse processes as rapid growth and change of direction in pollen tubes [9], morphogenesis of epidermal pavement cells and stomata [10,11], generation of diffusion barriers in root endodermis [12,13], and cell wall strengthening at sites of infection [14]. Yet, despite the importance of extracellular structures in development and disease, the question of how cells decide when, where, and how these materials should be produced, deposited, and specifically assembled or modified is far from being understood in any system.

Pollen presents a unique and powerful model for studying how controlled formation and deposition of extracellular structures are achieved. Pollen grains are surrounded by a complex extracellular structure, pollen wall exine, which assembles into intricate 3D patterns exhibiting enormous morphological diversity across species, yet very conserved within a species. What genetic and developmental programs control the precise formation of pollen surface patterns is completely unknown.

In most plant species, the pollen surface has characteristic areas called apertures, which either lack exine altogether or have decreased exine deposition [15,16]. They are often used as sites for pollen tube exit during pollen germination and as sites that help accommodate changes in volume during pollen dehydration or rehydration [16–20]. Aperture characteristics, such as number, positions, and morphology, are species-specific and, together with exine patterns, contribute to patterns on pollen surface. Arabidopsis pollen, similar to pollen of many other eudicot species, has three long and narrow apertures placed like equidistant meridians around the pollen equator. Their formation is quite precise: essentially all pollen grains in the wild-type Columbia accession of *Arabidopsis thaliana* develop three equidistantly placed apertures.

The existence of apertures indicates that, in a given species, certain areas on the pollen surface are specified in a different way from the rest of the surface and that these differences are reliably recognized by the exine deposition machinery so that, in the mature pollen, exine will be absent or reduced at these sites. Studying how pollen generates and defines regions destined to become apertures and to be free of exine might therefore provide a valuable insight into how cells form distinct extracellular domains and how they prevent deposition of extracellular materials at specific areas.

Apertures start forming at the time in pollen development when four microspores–the products of male meiosis–are still held together in a tetrad and are surrounded and separated from each other by callose walls [21–23]. Soon afterwards, callose walls are degraded and microspores separate and develop into individual pollen grains. Eighty years ago, based on the analysis of species in which separation of microspores does not happen and mature pollen is released as tetrads, Roger P. Wodehouse proposed an elegant model for how aperture positions might be specified. According to this model, in the eudicot species with three equatorial apertures (like in Arabidopsis), apertures form at the points of last contact between the sister microspores at the end of meiotic cytokinesis [16]. Wodehouse hypothesized that the geometrical arrangement of microspores in a tetrad (e.g. tetrahedral), which dictates the number of contact points, will correspondingly determine the number of developing apertures and will cause apertures on one microspore to be aligned with apertures in its sisters. This model suggested that cues for aperture placement arise as a result of cytokinesis and that last-contact points serve as aperture initiation sites.

In addition to the Wodehouse explanation of how pollen might develop a certain number of apertures, studies on pollen morphology in several plant species led to anecdotal reports suggesting that aperture number exhibits some positive correlation with pollen ploidy [24–29].

Although such observations and hypotheses had been made and cited by botanists, palynologists, and evolutionary biologists throughout the years, in the absence of a genetically tractable system that is amenable to extensive experimental manipulation, the predictions that ploidy, cytokinesis, and the geometric arrangement of microspores might be involved in aperture formation remained largely untested.

Recent discoveries of mutants in Arabidopsis that lack or have abnormal number of apertures [21,30], as well as of mutants with defective meiosis leading to abnormal geometric arrangement of meiotic products and/or abnormal pollen ploidy [31,32], allowed us to finally test in this species both the Wodehouse model and the effect of ploidy on aperture patterning. Through a systematic analysis and genetic manipulations of several Arabidopsis mutants and accessions with abnormal ploidy and/or abnormal geometry of meiotic products, we demonstrate here that a mechanism sensitive to pollen ploidy, rather than positions of the last-contact points or the 3D arrangement of post-meiotic microspores, acts as the major, although not the sole, determinant of aperture patterns and that this mechanism directs the aperture factor INP1 to specific positions on the microspore surface where apertures will be formed.

## Results

### *lsq* plants are tetraploid and produce diploid pollen

**Fig 1:**
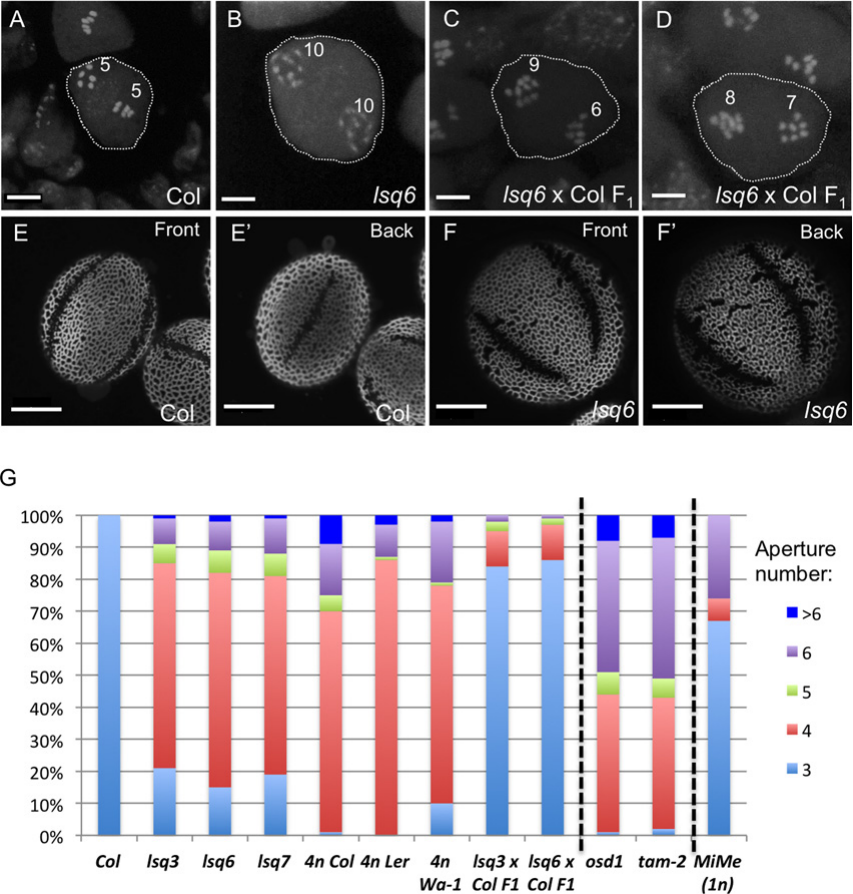
*lsq* lines are tetraploid and have ectopic pollen apertures. (A-D) Karyotypes of microspore mother cells undergoing meiosis I. (A) A wild-type Columbia plant (2n); (B) *lsq6* plant (4n); (C, D) F_1_ progeny of a cross between Columbia and *lsq6* (3n). (E-F’) Pollen with 3 apertures from wild-type Columbia (E, E’) and with four apertures from *lsq6* (F, F’). Front and back views of the same pollen grains are shown. Scale bars = 5 μm in (A-D) and 10 μm in (E-F’). (G) Percentages of pollen grains having indicated number of apertures in pollen populations from wild-type Col plants and from different mutants and accessions used in this study. n = 150–250 pollen grains.

Previously, in a forward genetic screen for mutants with defective pollen exine we discovered several *lsq* plants, which produced pollen with an abnormal number of apertures on their surfaces [30]. In addition to this, pollen of these plants was larger than normal (Figs 1 and S1) and, when dehydrated, often had rectangular shape, unlike the normal oval shape of the dehydrated wild-type pollen [30]. This phenotype was easily recognizable at the dissecting-microscope level and gave the name to these plants–*l**arge and* *sq**uare pollen*. To understand the nature of these defects, we concentrated on three of these mutants, *lsq3*, *lsq6*, and *lsq7*. The increased size of these pollen grains prompted us to examine their ploidy level, as ploidy often positively correlates with cell size [33,34] and, in particular, with pollen size [35,36]. We examined the karyotypes of the *lsq* pollen mother cells undergoing meiosis I and of somatic elongated cells in anther filaments and found that both types of cells had twenty chromosomes (Figs 1A and 1B and S2), twice the normal number (2n = 10), indicating that *lsq* plants were tetraploid and produced diploid, rather than normal haploid, pollen. The F_1_ progeny from the cross between *lsq* and diploid wild-type Columbia (Col) exhibited unequal segregation of 15 chromosomes in male meiosis I (Fig 1C and 1D), consistent with the expected triploidy in the F_1_ generation.

### Tetraploid Arabidopsis plants predominantly produce pollen with four apertures

**Fig 2:**
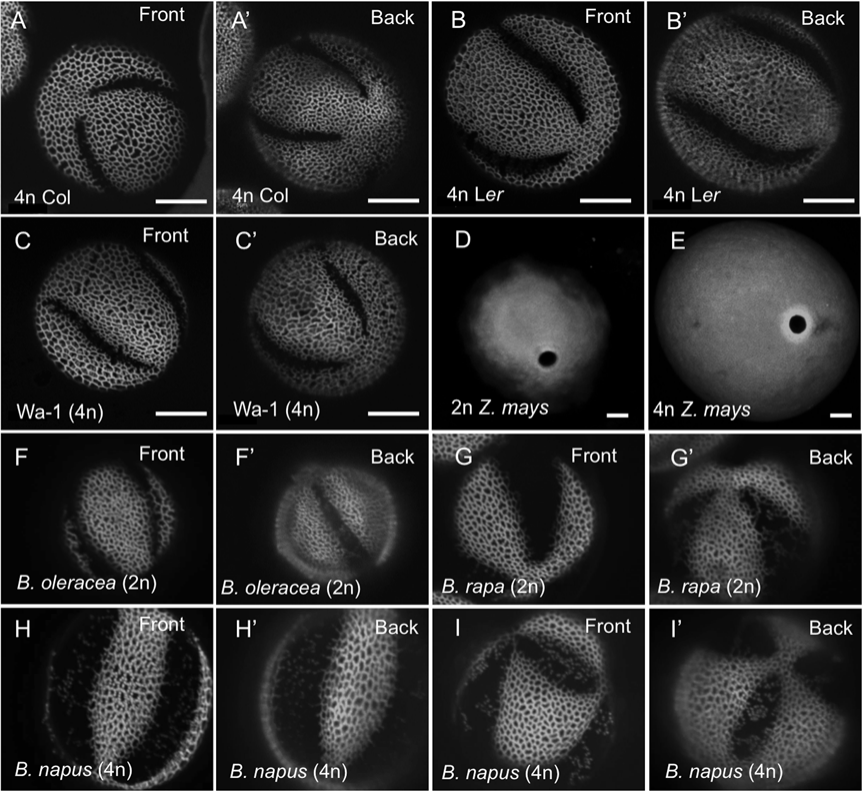
Changes in pollen ploidy correlate with changes in pollen aperture numbers in different Arabidopsis ecotypes and in *Brassica* species, but not in maize. (A-C’) Front and back views of pollen grains with four apertures from tetraploid Columbia (A, A’), tetraploid Landsberg *erecta* (B, B’), and tetraploid Warschau (C, C’) accessions of Arabidopsis. (D, E) Pollen from both diploid (D) and tetraploid (E) maize has a single pore-like aperture. Scale bars = 10 μm. (F-I’) Pollen from a diploid *B*. *oleracea* (F, F’) and *B*. *rapa* species has 3 apertures, whereas pollen from their allotetraploid hybrid *B*. *napus* often has more than 3 apertures. Front and back views of *B*. *napus* pollen with four apertures (H, H’) and with six apertures (I, I’). Plant ploidy is indicated on all images.

Using confocal microscopy, we determined the number of apertures in pollen produced by the three *lsq* lines and found that, although the numbers varied between individual pollen grains, most (~65%) of the pollen in these populations had four equatorial apertures (y1F and 1G). To check if the change from three to predominantly four apertures was possibly caused by the changes in ploidy, we determined the number of apertures produced by several tetraploid Arabidopsis accessions available from the Arabidopsis Biological Resource Center: 4n Col, 4n Landsberg *erecta* (L*er*), and the natural 4n accession Warschau (Wa-1). All of these plants produced pollen that had larger size, *lsq*-like morphology and mostly four apertures (Figs 1G, 2A–2C’ and S1), strongly suggesting that indeed higher ploidy, rather than potential mutations, was responsible for the formation of more than the normal complement of apertures in the *lsq* plants. In contrast, pollen from the triploid F_1_ plants usually had 3 apertures (Figs 1G and S3), and was close to the wild-type pollen in size and appearance.

To see if pollen from other *Brassicaceae* species also exhibited positive correlation between higher ploidy and higher aperture number, we examined pollen from the diploid Brassica species *B*. *oleracea* and *B*. *rapa*, as well as from their allotetraploid hybrid *Brassica napus*. While both *B*. *oleracea* and *B*. *rapa* consistently produced triaperturate pollen (Fig 2F–2G’), the *B*. *napus* pollen often had more than three apertures (Fig 2H–2I’). Interestingly, in pollen of a grass, maize *Zea mays*, we did not observe a similar correlation between the ploidy and the number of apertures. While pollen from 4n maize was larger than pollen from 2n maize, it still developed a single pore-like aperture (Fig 2D and 2E).

### *lsq* plants form normal tetrahedral tetrads

The Wodehouse model of aperture formation proposed that in plants like Arabidopsis, which have three equidistant apertures and a tetrahedral arrangement of microspores in tetrads, apertures are formed at the last points of contact persisting between the cytoplasm of future microspores at the end of simultaneous cytokinesis, during which six cell walls are formed from the periphery to the center [16]. Four microspores arranged in a tetrahedral tetrad have six contact points and form 12 apertures (3 per microspore) that develop in the vicinity of these points. The long-standing Wodehouse model agrees well with the situation observed in the wild-type Arabidopsis, where apertures can be already observed at the tetrad stage and are visible as half-apertures close to the center of the planes dividing the sister microspores (S1 Movie).

**Fig 3:**
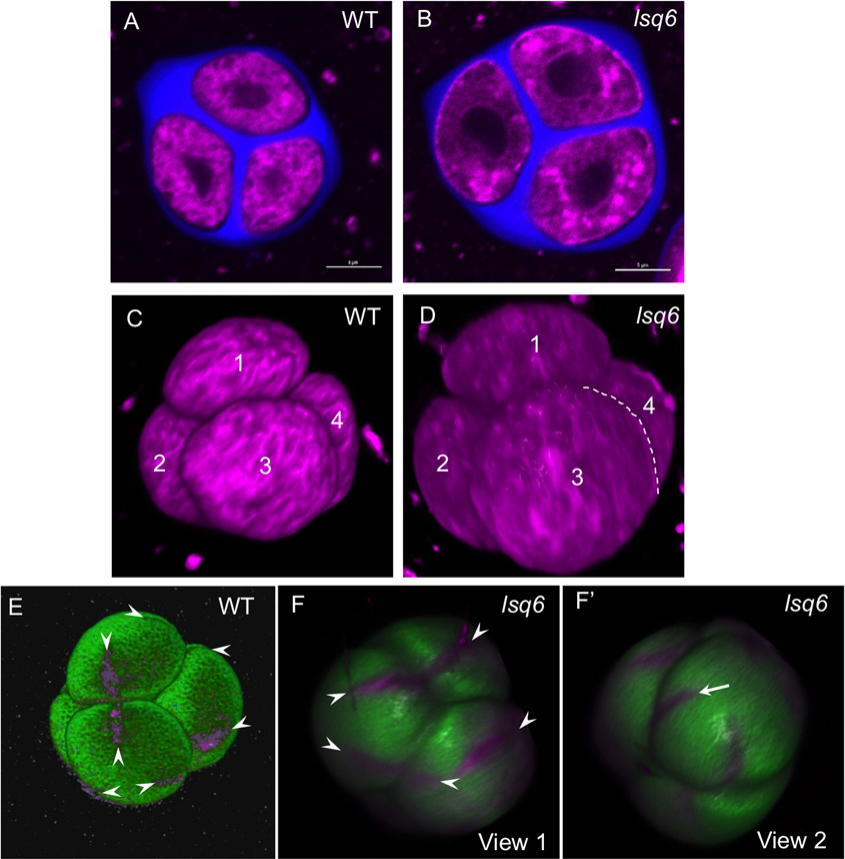
*lsq* plants have normal tetrahedral arrangement of microspores in a tetrad. Some of the developing apertures in *lsq6* tetrads are not aligned with any apertures in sister microspores. (A-D) Tetrads from the wild-type (A, C) and *lsq6* (B, D) plants exhibit similar tetrahedral morphology. (A, B) Single optical sections. Membrane structures are stained with CellMask Deep Red (magenta) and callose walls are stained with calcofluor white (blue). Scale bars = 5 μm. (C, D) 3-D reconstructions from confocal z-stacks of the tetrads shown in (A) and (B). Each of the four microspores in a tetrad is labeled with a number. (E-F’) 3-D reconstruction and surface rendering from confocal z-stacks of a wild-type (*INP1pr*:*INP1-YFP*) (E) and a *lsq6* (F, F’) tetrad of microspores allows visualizing apertures developing on microspore surfaces (magenta). Microspore surfaces were transiently labeled with DAPI (green) and rendered with IMARIS (E) or Nikon Elements (F, F’). Developing apertures (indicated by arrowheads) are visible due to underlying INP1-YFP fluorescence (E, magenta) or with the help of the membrane stain CellMask Deep Red (F, F’, magenta). (F) A view of a *lsq6* tetrad that shows alignment between apertures on sister microspores (arrowheads). (F’) A different view of the same tetrad that shows an aperture (arrow) not aligned with any apertures in a sister microspore. See also S1 and S2 Movies.

**Fig 4:**
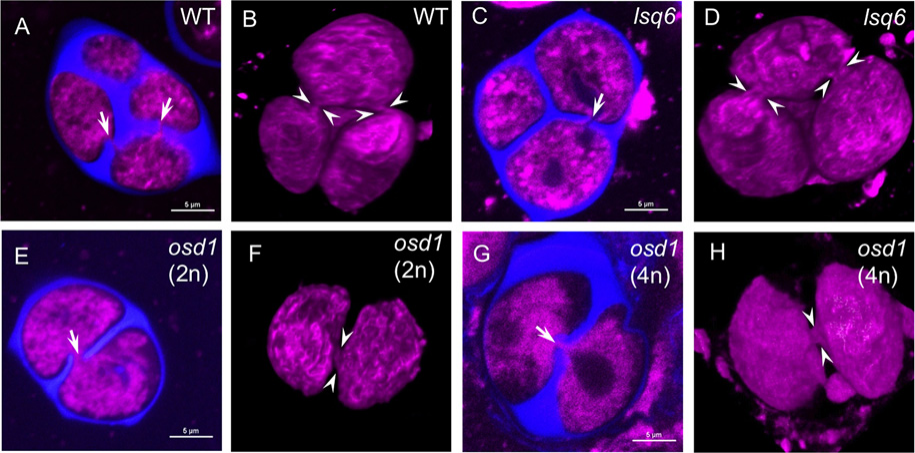
Positions of the last points of contact in tetrads of wild type and *lsq6* and in dyads of 2n and 4n *osd1* plants. (A, C, E, G) Single optical sections through tetrads and dyads. Gaps in callose walls and last points of contact are labeled with arrows. Membrane structures are stained with CellMask Deep Red (magenta) and callose walls are stained with calcofluor white (blue). Scale bars = 5 μm. (B, D, F, H) 3-D reconstructions from confocal z-stacks of the tetrads and dyads shown in (A, C, E, G). Positions of cytoplasmic bridges at the last points of contact are indicated by arrowheads.

To check if the abnormal number of apertures in the *lsq* pollen was caused by the abnormal development or arrangement of tetrads of microspores, we compared the morphology of the *lsq* tetrads with the wild-type tetrads (Fig 3A–3D). As expected, microspores in the *lsq* tetrads were larger, but otherwise they exhibited normal tetrahedral conformation, indicating that the tetrahedral arrangement of microspores in a tetrad is not sufficient to specify formation of three apertures. We also checked if positions of the last points of contact were changed in the *lsq* tetrads compared to the wild-type tetrads. We found that similar to wild type, *lsq* tetrads developed points of last contact close to the center of the division plane formed by the callose wall (Fig 4A–4D).

### Accommodation of the fourth aperture in the 3D context of a tetrahedral tetrad

Arrangement of four wild-type haploid Arabidopsis microspores in a tetrahedral tetrad generates tri-radial symmetry in each microspore and creates three natural positions for equidistant apertures. Indeed, these positions close to the middle of the division planes that separate microspores—or sites very close by—are used for aperture formation in the wild-type tetrads: each aperture in each microspore aligns with an aperture in a sister microspore (Fig 3E and S1 Movie). Therefore, it is intriguing how the appearance of the fourth apertures in 2n *lsq* microspores is accommodated in the 3D context of a tetrahedral tetrad. To understand how four apertures on a *lsq* microspore are placed in relation to apertures in its sister microspores, we imaged and 3D-reconstructed tetrads of 4n *lsq* plants by staining microspore surfaces with DAPI, which transiently stains pre-exine materials in some tetrads and allows visualization of the DAPI-negative aperture positions [21]. We found that in the 4n tetrads at least one microspore had all four apertures aligned with apertures in other three microspores, whereas other microspores usually had 1 to 2 apertures that were not aligned with their counterparts in sister microspores (Fig 3F and 3F’, S2 Movie). This suggests that a close contact and alignment between developing apertures in sister microspores is not required for aperture formation and that apertures on a given microspore can be positioned independently of apertures on its sisters.

### Aperture formation is not determined by the positions of last-contact points between microspores in cytokinesis

**Fig 5:**
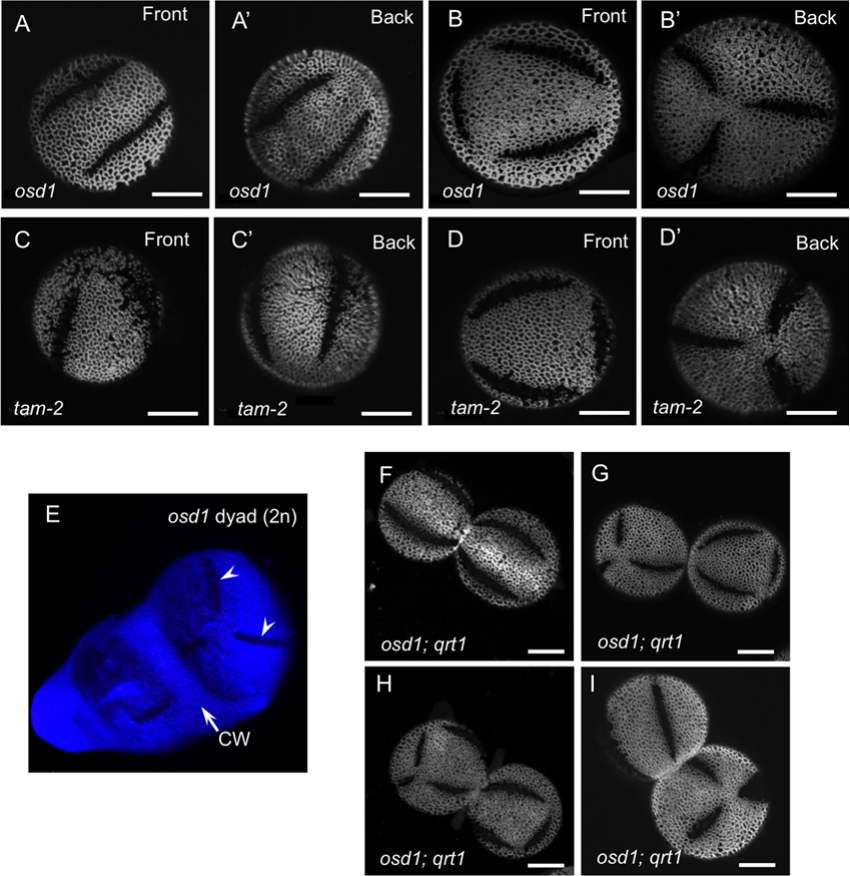
Diploid pollen in *osd1* and *tam-2* mutants has predominantly four or six apertures, some of which develop without any contact with intersporal callose wall. (A, B’) Front and back views of *osd1* diploid pollen with four (A, A’) or six (B, B’) apertures. (C-D’) Front and back views of *tam-2* diploid pollen with four (C, C’) or six (D, D’) apertures. (E) 3D reconstruction of a diploid *osd1* dyad at the stage when developing apertures become visible on the surfaces of microspores. Two apertures that develop at the distal side of one of the microspores, away from the intersporal callose wall (CW), are indicated by arrowheads. Microspore surfaces were stained with DAPI and callose wall was stained with calcofluor white (both blue). (F-I) Dyads of mature pollen from the *osd1; qrt1* plants showing examples of dyads with 4/4 (F), 6/6 (G), 8/6 (H), and 4/6 (I) aperture configurations. Scale bars = 10 μm.

In addition to being produced by tetraploid plants, diploid pollen can arise in diploid plants as a result of abnormal meiosis. Arabidopsis *osd1* and *tam-2* mutants have meiotic defects, leading to omission of the second meiotic division and to the formation of dyads, and not tetrads, of diploid microspores [31,32]. Formation of the single cell wall separating the products of meiosis in such dyads results in generation of just a single last-contact point, close to the center of the division plane between microspores (Fig 4E and 4F). We, therefore, asked whether the single contact point developing in *osd1* and *tam-2* dyads would lead to the formation of a single aperture per microspore. We examined the aperture number in the pollen produced by the *osd1* and *tam-2* plants and found that this was not the case. Similarly to the 2n *lsq* pollen, 2n *osd1* and *tam-2* pollen grains were larger than normal (S1 Fig) and had not one, but usually more than three apertures on their surface (Figs 1G and 5A–5D’). However, the distribution of aperture numbers within these pollen populations was somewhat different from *lsq*: unlike the predominantly four apertures in the *lsq* pollen, *tam-2* and *osd1* commonly had approximately equal ratio (~40% each) of pollen with either four or six apertures (Fig 1G), with four apertures arranged around the equator of a pollen grain, and six apertures usually forming the edges of an imaginary tetrahedron inscribed into a pollen grain (Fig 5A–5D’). These results clearly demonstrate that the last-contact points between microspores do not determine the number and positions of apertures on pollen surface. However, the observed difference between the aperture numbers in populations of 2n pollen generated by two different mechanisms (via tetrad vs. via dyad) suggests that the type of microspore arrangement after cytokinesis still has some effect on aperture placement. As an additional confirmation for positions of apertures being independent of the positions of the last-contact points, we were able to occasionally catch *osd1* dyads at the stage when apertures were already visible on microspore surfaces. An image of such a dyad in Fig 5E clearly shows two apertures developing in the distal part of a microspore, without any contact with the intersporal callose wall.

The approximately 1:1 ratio of pollen with four and six apertures produced by the *osd1* and *tam-2* mutants led us to hypothesize that this could be due to dyads giving rise predominantly to one grain with four apertures and another with six apertures. To test this hypothesis, we created *osd1; qrt1* double mutants, in which the *qrt1* mutation prevented separation of meiotic products [37], leading to the release of mature pollen grains as dyads. Analysis of apertures in such dyads, however, demonstrated that our hypothesis was not correct, as most of the mature pollen dyads exhibited a symmetric distribution of aperture numbers in their constituents (Fig 5E–5G): e.g. out of 116 pollen dyads, 37% had 4 apertures in both grains (4/4 configuration), 34% had a 6/6 configuration, and 6% had closely related 6/8, 6/7, 7/8, or 8/8 configurations. In the cases of more than six apertures in a grain, there were usually six long apertures distributed along the edges of a tetrahedron, similar to the hexaperturate configuration described above, as well as one or two short apertures, suggesting that the 7- or 8-aperture configurations were the derivatives of the 6-aperture configurations. The asymmetric 4/6-aperture dyads, although observed in this population (Fig 5H), were less frequent (16%, n = 116).

### Pollen with normal ploidy generated through dyad formation has predominantly normal number of apertures

**Fig 6:**
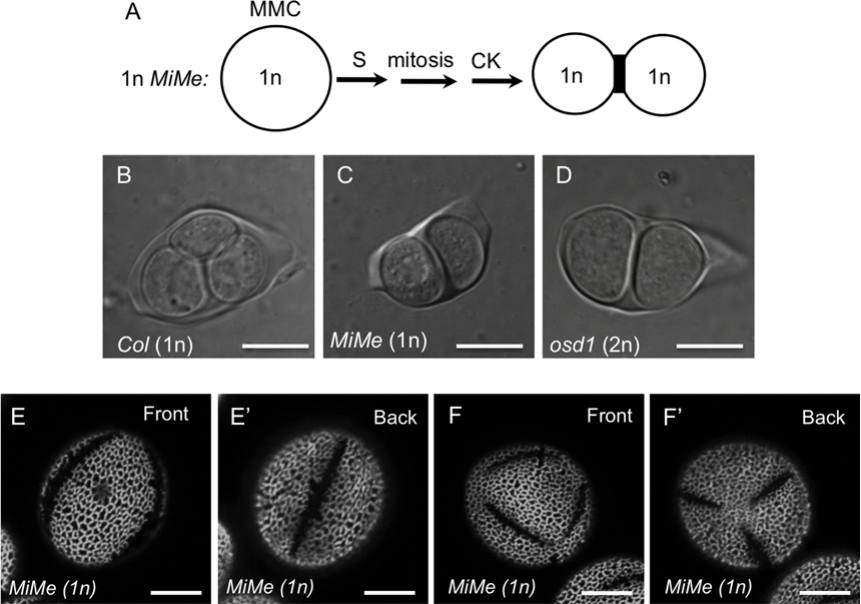
The majority of haploid pollen in 1n *MiMe* plants has three normal apertures. (A) A diagram of microsporogenesis that occurs in the haploid *MiMe* plants and leads to the formation of haploid dyads of microspores. MMC–microspore mother cell, S–DNA synthesis, CK–cytokinesis. (B-D) Haploid *MiMe* microspores in a dyad (C) are similar in size to haploid wild-type microspores in a tetrad (Col, B) and are smaller than diploid *osd1* microspores in a dyad (D). (E-E’) 67% of pollen produced by the haploid *MiMe* plants had three normally arranged apertures. (F-F’) In ~ 25% of cases, 1n *MiMe* pollen had six apertures. Scale bars = 10 μm.

To look further into the unexpected result that the last-contact points in Arabidopsis do not specify aperture number and positions, we attempted to reduce the number of variables in our experimental system by keeping pollen ploidy normal (1n) and modifying only the arrangement of microspores at the end of cytokinesis (dyads vs. tetrads). To achieve this, we used *MiMe* plants, in which three mutations convert pollen meiosis into a mitosis-like division [31]. By crossing *MiMe* triple heterozygotes with a haploid inducer line [38], we generated haploid *MiMe* plants. These 1n plants produced 1n pollen through a mitosis-like division followed by dyad formation, generation of a single cleavage wall, and a single contact point (Fig 6A and 6C). This dyad-generated haploid pollen had normal size (S1 Fig) and, surprisingly, most of the grains (67%, n = 200) developed three normally placed apertures (Figs 1G and 6E and 6E’). This result clearly confirms that contact points do not dictate the number and positions of apertures. It also indicates that 1) the tetrahedral microspore arrangement is not necessary for the development of three normal equatorial apertures, and 2) a ploidy-related or ploidy-sensitive mechanism is likely to be the most important in determining aperture number and positions.

Interestingly, however, in this population of the haploid *MiMe* pollen grains the next most commonly observed aperture pattern (~25% of pollen grains; n = 200) was six apertures arranged along the edges of a tetrahedron (Figs 1G and 6F and 6F’). This suggests that ploidy or characteristics related to ploidy (e.g. cell size), although important, do not act as the absolute determinants of aperture number.

### Pollen ploidy higher than 2n leads to further increase in aperture number and to changes in aperture morphology

**Fig 7:**
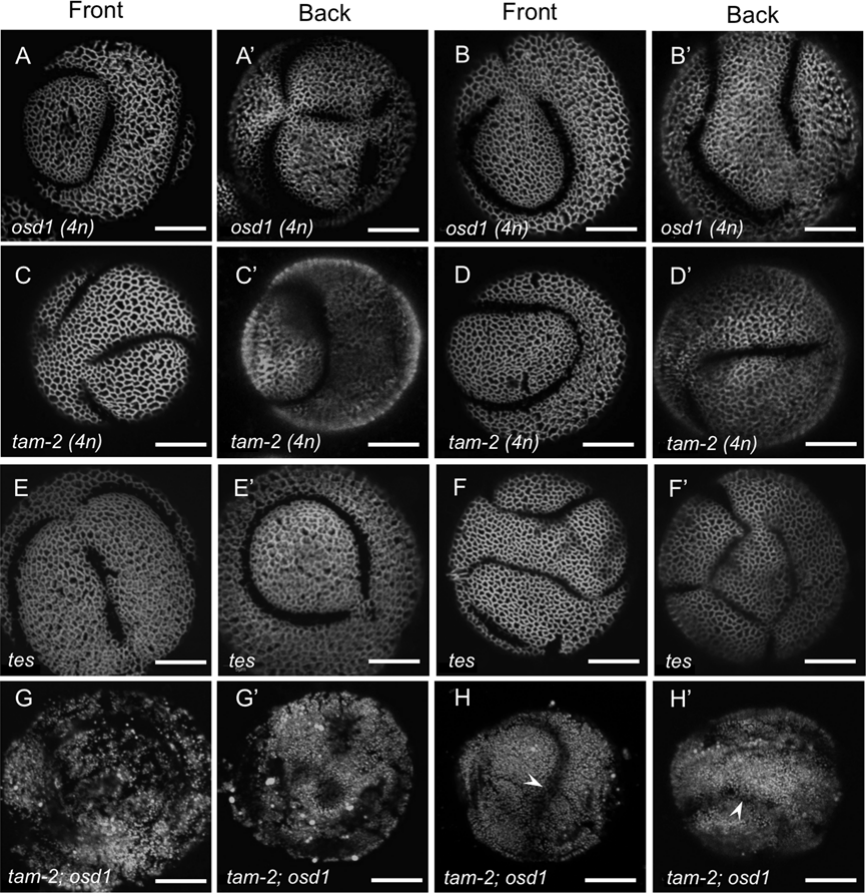
Pollen ploidy higher than 2n is accompanied by changes in aperture morphology. (A-F’) Ring-shaped apertures are common in tetraploid pollen from *osd1* (A-B’), *tam-2* (C- D’), and *tes* (E-F’). Front and back views are shown for each pollen grain (e.g. A and A’). (G-H’) Tetraploid pollen from *tam-2; osd1* mutants often has abnormal exine patterns that make recognizing apertures difficult. Still, ring-shaped apertures are sometimes visible in *tam-2; osd1* pollen (H, H’, arrowheads). Scale bars = 10 μm.

In addition to having diploid pollen, *osd1* and *tam-2* plants almost always produce diploid egg cells [31,32], thereby allowing generation of higher-ploidy progeny through self-pollination. We took advantage of this to determine whether further increase in ploidy levels will cause additional changes in aperture characteristics. The 4n pollen grains produced by the 4n *osd1* and *tam-2* plants were larger than the 2n pollen (S1 Fig) and commonly had unusual ring-like apertures (Fig 7A–7D’) with variable morphology, number and positions. As some of these pollen grains had apertures that formed incompletely closed rings (Panel A in S4 Fig), existence of these ring-like structures may be explained by the initiation of multiple ectopic apertures, which, as the apertures elongate, get connected into the ring-like structures. Similar to the *osd1* and *tam-2* diploid pollen, this tetraploid pollen developed via a dyad stage, in which microspores were separated by a single wall and formed a single last-contact point close to the center of the dividing wall (Fig 4G and 4H).

Interestingly, the ring-like aperture phenotypes produced in the dyad-derived 4n *osd1* and *tam-2* pollen were strongly reminiscent of the phenotypes of the Arabidopsis *tetraspore* (*tes*)/*stud* mutants [39,40] (Fig 7E–7F’). During microsporogenesis in *tes*, both nuclear meiotic divisions occur normally, but the lack of cytokinesis leads to the generation of multi-nuclear syncytial pollen, which, in effect, is a tetraploid monad [39,40]. Similarities between the apertures in the 4n *tes* pollen produced in the absence of cytokinesis through a monad-like microspore and the 4n *osd1* or *tam-2* pollen produced via a dyad formation (Fig 7A–7F’) demonstrate that similar aperture phenotypes can be obtained in pollen of similar ploidy, independently of cytokinesis and of the microspore arrangement after cell division. This conclusion is further supported by the overall aperture similarity between the 2n *lsq* pollen that is tetrad-derived and the 2n dyad-generated *osd1* or *tam-2* pollen. In addition to the 2n pollen and 4n pollen produced through various mechanisms, we also examined the 3n pollen produced by hexaploid Arabidopsis plants through a normal meiotic division (plants were generated by treating with colchicine the 3n F_1_ progeny from the cross between a diploid and a tetraploid plant). These pollen grains usually had more than 4 apertures and, similarly to the 4n pollen, often developed ring-like apertures (Panels B-B’ in S4 Fig), indicating that there must be threshold levels of ploidy associated with certain aperture numbers: e. g., 1–1.5n pollen has mostly 3 apertures, 2n pollen has mostly 4–6 apertures, and pollen of 3n or higher ploidy often has ring-like apertures.

### The double *tam-2; osd1* mutant has severely disrupted exine and no recognizable apertures

The *tam-2; osd1* diploid double mutant lacks both rounds of nuclear divisions, as well as cytokinesis, during male meiosis [32], and therefore produces an equivalent of 4n pollen grains through a different mechanism from the ones described above. We used the *tam-2; osd1* double mutant to check if its pollen will develop apertures similar to ring-like apertures in 4n *osd1*, *tam-2* and *tes* pollen or whether the absence of meiotic nuclear division affects aperture placement.

The double *tam-2; osd1* mutant has strong reduction in fertility and in the number of pollen grains that it produces [32]. Still, we were able to recover some *tam-2; osd1* pollen grains. Interestingly, unlike the pollen in all previously analyzed mutants, these pollen grains had severely disrupted exine patterns (Fig 7G and 7H’) and, likely because of that, usually had no recognizable apertures. Occasionally, however, ring-shaped apertures were observed in *tam-2; osd1* pollen (Fig 7H and 7H’). As exine patterns were normal in *osd1* lacking meiosis II and in *tes* lacking cytokinesis, abnormal exine formation in *tam-2; osd1* suggests a possibility that meiosis I might be necessary for production or distribution of factors that specify exine patterns.

### The higher number of apertures developing in the higher-ploidy pollen is not caused by the increased levels of the aperture factor INP1

We demonstrated that aperture number has strong positive correlation with the increase in pollen ploidy. Increased ploidy leads to the increase of DNA-related factors, such as chromosome number, gene dosage, and, possibly, levels of gene products. It also commonly leads to the increased cell size [33–36,41]. So, in theory, formation of ectopic apertures in pollen with higher ploidy can either be caused by the increased levels of aperture-promoting factors or by the mechanism that is linked to sensing microspore dimensions.

The only molecular player currently known to be involved in pollen aperture formation is the Arabidopsis gene *INAPERTURATE POLLEN1* (*INP1*), whose protein product normally localizes to three areas close to microspore surface where apertures will form [21]. Inactivation of INP1 causes the complete lack of pollen apertures, while reduction in the INP1 expression levels leads to the development of apertures that are shorter than normal [21]. These data suggest that apertures are sensitive to the *INP1* levels and that *INP1* might be susceptible to the gene-dosage effects. However, the very low and transient levels of *INP1* expression, as well as difficulties in identifying *INP1*-expressing anther stages based on a floral bud/anther morphology, pose a significant challenge in reliably measuring the amounts of endogenous *INP1* [21]. Therefore, to overcome this problem and see if the effects of ploidy on aperture number might be caused by changes in the *INP1* gene dosage (and by the related changes in the *INP1* product levels), we took advantage of the two previously characterized *INP1pr*:*INP1-myc; inp1* transgenic lines that have lower-than-normal levels of *INP1* expression and produce pollen with short or medium-size apertures [21]. By treating these plants with colchicine, we generated tetraploid versions of these lines, producing diploid pollen. We then assessed the number of apertures in these pollen grains.

**Fig 8:**
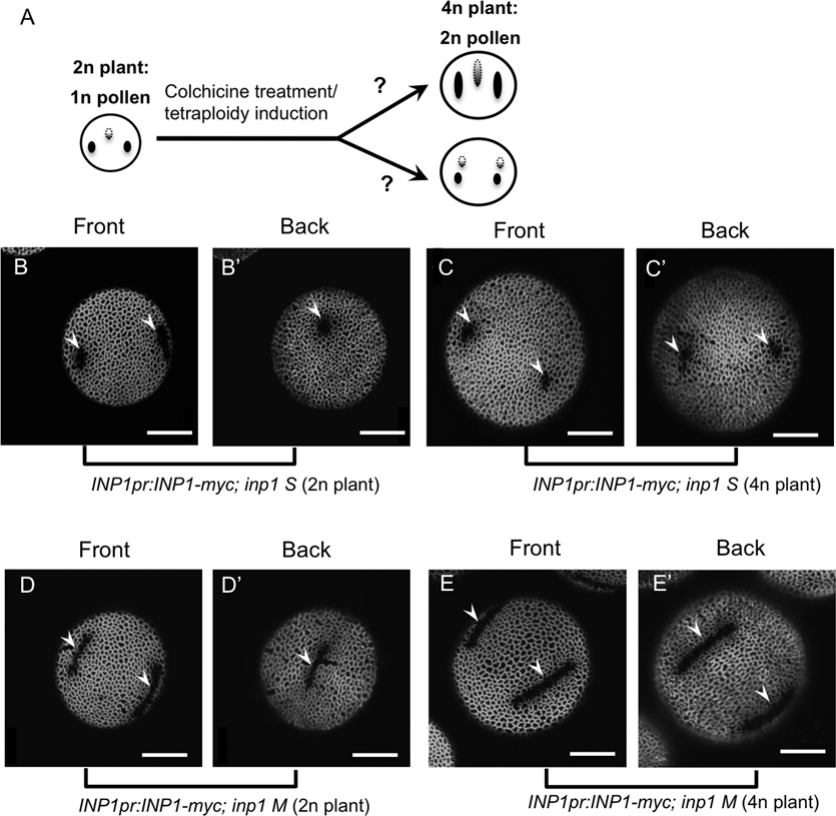
Pollen grains of the 4n plants generated from the 2n lines with reduced levels of *INP1* expression and shortened apertures develop four shortened apertures. (A) Schematic description of the experiment to distinguish between possible mechanisms involved in specifying aperture number. Tetraploid plants generated from the diploid short-aperture line by colchicine treatment are expected to produce 2n pollen with 3 longer apertures if INP1 level is the factor that determines the number of apertures. 2n pollen with 4 short apertures is expected if other factors (e.g. levels of another gene or changes in cell geometry) dictate aperture number. (B-C’) An *INP1pr*::*INP1-myc; inp1 S* line produces three short apertures in haploid pollen (B-B’) and four short apertures in diploid pollen (C-C’). An *INP1pr*::*INP1-myc; inp1 M* line produces three medium-size apertures in haploid pollen (D-D’) and four medium-size apertures in diploid pollen (E-E’). Apertures are indicated by arrowheads. Scale bars = 10 μm.

We reasoned that if ectopic apertures in the 4n plants with normal *INP1* (e.g. in *lsq* or 4n Col) are caused by the increased *INP1* product levels as the result of the increased gene dosage, then the increased gene dosage in the 4n *INP1pr*:*INP1-myc; inp1* plants (originating from the lines with reduced *INP1* levels and shortened apertures) should first lead to an increase in aperture length, before the formation of ectopic apertures (Fig 8A). If, however, the ectopic apertures in 2n pollen result from the changed geometry of the cells (e.g. larger microspores have intrinsic propensity to generate four aperture-initiation sites) or from the increased levels of unknown factors that localize to four positions in 2n microspores and attract INP1, then pollen with four short apertures would form in the 4n *INP1pr*:*INP1-myc; inp1* plants (Fig 8A).

We found that, similarly to the pollen of regular tetraploid plants, both 4n *INP1pr*:*INP1-myc; inp1* lines generated pollen with four apertures. Aperture size in these 2n pollen grains was similar to the size of apertures in the parental lines–short for the short-aperture parental line and medium for the medium-aperture parental line (Fig 8B–8E’). These data indicate that ectopic apertures in 2n pollen are caused not by the increase in the *INP1* levels, but by another mechanism, such as increased cell size or, potentially, increased levels of unknown gene products.

**Fig 9:**
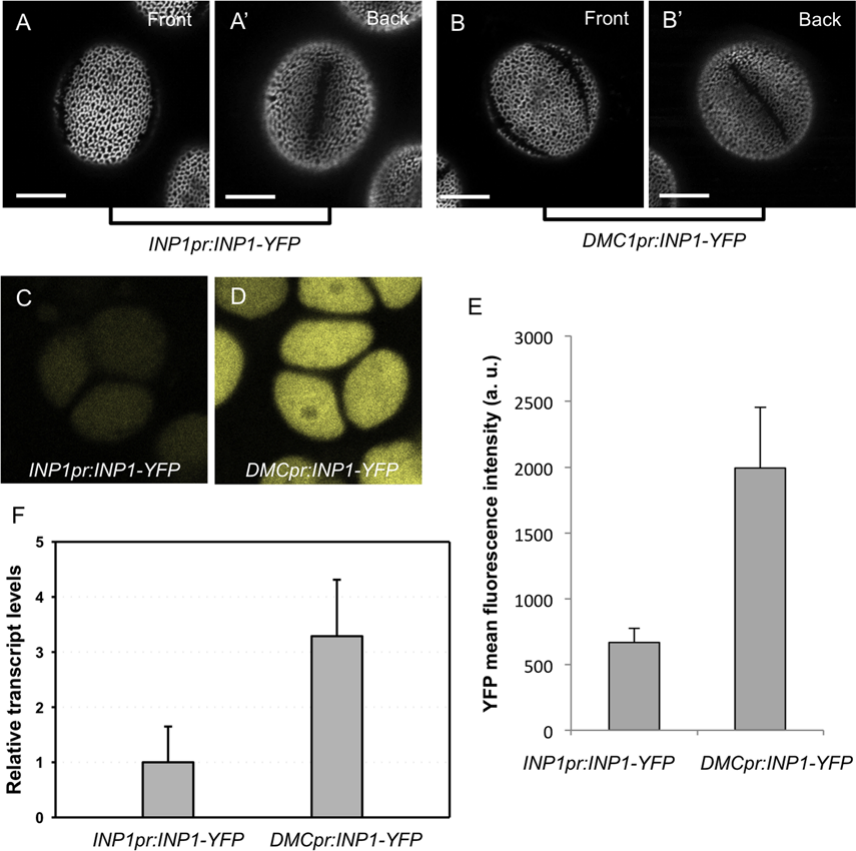
Higher levels of INP1 expression do not result in increased number of apertures. (A-B’) Front and back views of pollen from the *INP1pr*:*INP1-YFP; inp1* line (A, A’) and the *DMC1pr*:*INP1-YFP; inp1* line (B, B). Scale bars = 10 μm. (C, D) Representative images of optical sections through tetrads that were used to measure the mean intensity of diffused YFP fluorescence in the *INP1pr*:*INP1-YFP; inp1* (C) and the *DMC1pr*:*INP1-YFP; inp1* (D) lines. (E) Quantification of mean YFP fluorescence in *INP1pr*:*INP1-YFP* and *DMC1pr*:*INP1-YFP* tetrad-stage microspores (a.u. = arbitrary units). Error bars indicate SD. (F) *INP1-YFP* transcript levels were measured by qRT-PCR and normalized to the levels of the endogenous *inp1-1* transcript. Error bars indicate SE (n = 4 biological replicates).

Additional support for the notion that higher aperture numbers are not caused by the increased INP1 levels was obtained by comparing aperture numbers and the INP1 expression in lines containing *INP1-YFP* transgenes driven either by the *INP1* native promoter [21] or by the *DMC1* promoter, which is strongly expressed in the sporogenous layer of anthers [42]. Expression of either transgene in the *inp1* mutant is sufficient to restore the apertures (Fig 9A–9B’). However, the *DMC1pr*-driven construct is consistently expressed at levels that are 3- to 5-fold higher comparing to the *INP1pr*-driven construct, as judged by quantitative RT-PCR and by measuring YFP fluorescence of tetrads (Fig 9C–9F). Despite the much stronger INP1 expression in the *DMC1pr*:*INP1-YFP* plants, the pollen grains produced by these plants still formed only three apertures (Fig 9A–9B’).

### INP1 is associated with developing ectopic apertures and is necessary for their formation

**Fig 10:**
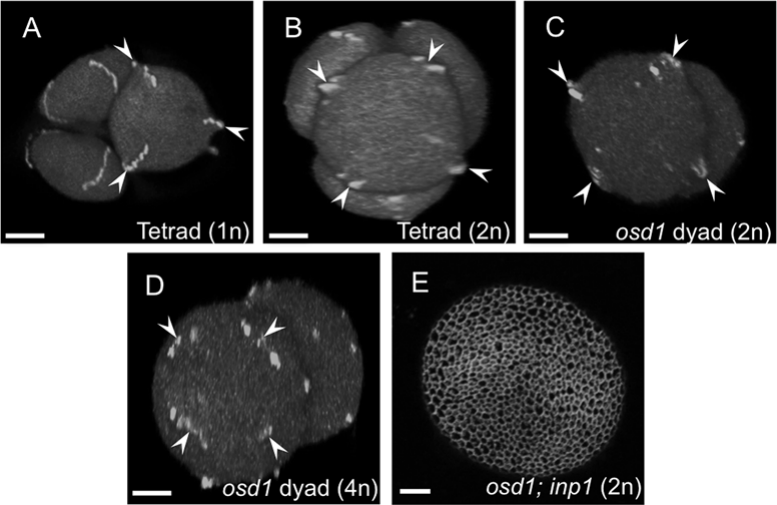
INP1 localizes to the positions of ectopic apertures and is required for their formation. (A-D) Tetrads or dyads of microspores expressing *INP1pr*:*INP1-YFP* viewed from the distal pole of a microspore. Ploidy of a microspore is indicated. INP1-YFP forms three punctate lines per microspore in haploid tetrads of wild-type 2n plants (A), but forms ectopic lines (four or more) in tetrads of diploid microspores of 4n plants (B) or in diploid or tetraploid dyads of 2n (C) or 4n (D) *osd1* plants. Positions of INP1-YFP lines are indicated by arrowheads. Note the four lines of INP1-YFP puncta assembling into a ring-like structure on a distal surface of a microspore in the 4n *osd1* dyad (D, arrowheads). (E) Pollen grains in the *osd1; inp1* double mutant lack apertures. Scale bars = 5 μm.

We have previously demonstrated that in the normal haploid tetrads of microspores the INP1 protein is associated with developing apertures and forms three long and narrow lines of puncta close to the microspore surface, which mark positions of future apertures (Fig 10A) [21]. To check whether ectopic apertures get similarly pre-marked by the INP1 localization, we used the previously described *INP1pr*:*INP1-YFP* line [21]. Again, using colchicine treatment, we generated the 4n version of this transgenic line. In addition, we crossed this transgene with *osd1/+* to generate the 2n *osd1;INP1pr*:*INP1-YFP* plants. In the diploid tetrads and dyads that developed, respectively, in these two types of plants, INP1-YFP was found to form four or more lines of puncta at the microspore surface (Fig 10B and 10C), demonstrating that INP1 gets successfully delivered to the positions of future ectopic apertures. Moreover, in the 4n microspores generated by the 4n *osd1;INP1pr*:*INP1-YFP* plants, INP1-YFP puncta often formed ring-like structures, strongly reminiscent of the ring-shaped apertures that develop in the pollen of these plants (Fig 10D). These results demonstrate that INP1 gets delivered to the positions of ectopic apertures and pre-marks these sites.

To test if INP1 is necessary for the formation of ectopic apertures, we crossed the *inp1* mutant with *osd1/+* and examined pollen of the *inp1; osd1* double mutant. Similarly to the *inp1* pollen, the *inp1;osd1* pollen lacked apertures (Fig 10E), indicating that both normal and ectopic apertures depend on the presence of functional INP1 product.

## Discussion

How the intricate and complex geometric patterns develop on pollen surfaces is a fascinating and long-standing question in cell biology. Pollen apertures constitute a type of pollen-surface patterning elements that often develop with extraordinary species-specific precision in regard to their number, morphology, and position. The diversity of aperture patterns found in different species prompted the creation of models to explain how different patterns could be formed [16,43,44]. One of the primary components of these models is the progression of cytokinesis that leads to formation of callose walls separating microspores and to development of tetrads that can have different geometrical arrangements. The Wodehouse model was the first to suggest that the last contact points present between the microspores at the end of cytokinesis determine the position of the apertures on the pollen grain surface [16]. Later, Ressayer et al. extended this model to species with other types of apertures and different modes of cytokinesis and proposed that positions of the last-contact points, serving as spatial cues for determining sites for aperture formation, would depend on the type of cytokinesis (successive or simultaneous), direction of the intersporal wall formation (centrifugal or centripetal), and tetrad shape (tetrahedral, tetragonal/decussate, or rhomboidal) [44].

The fact that the Wodehouse model fits so well with the observed positions of apertures in multiple eudicot species, including the ones that shed mature pollen as tetrads and allow easy observations of the relative positions of apertures in a tetrad (e.g. many species in *Ericaceae*, www.paldat.org), made it very attractive and allowed it to guide the field for eighty years. We, for example, previously invoked this model as a possible explanation for the delivery of INP1 to its three highly specific positions in the wild-type Arabidopsis microspores and for the formation of short apertures close to the pollen equator in the lines in which levels of INP1 expression are reduced [21]. Similarly, others have used this model as an explanation for the existence of rare pollen morphs in Nicotiana species and for the unexpected presence of triaperturate pollen in an Arabidopsis mutant with abnormal cytokinesis [45,46]. Yet, so far, the support for the Wodehouse and related aperture formation models mostly came from studies that tried to correlate aperture patterns with cytokinesis variations by comparing either different plant species or common and rare pollen morphs of the same species [22,46]. None of these systems, however, allowed rigorous testing of the proposed models. The further complication of these studies is that apertures become visible only after completion of cytokinesis–thus, in the absence of live imaging of developing tetrads (which has not yet been done in any species), only one of the two characters (cytokinesis or apertures) can be observed in a given tetrad, often making direct correlations between the progression of cytokinesis and aperture formation impossible.

Here, in order to actually test the Wodehouse model, we took full advantage of genetic and molecular resources available in Arabidopsis, coupled with advanced imaging techniques. As a species with a tricolpate equatorial aperture pattern, simultaneous meiotic cytokinesis, centripetal cell plate formation, and tetrahedral arrangement of microspores in a tetrad, Arabidopsis is the classical Wodehouse case. Surprisingly, by perturbing the process of cytokinesis in Arabidopsis, we found that, contrary to the Wodehouse hypothesis, the geometric arrangement of microspores at the end of cytokinesis and the related number of contact points between sister microspores do not play a major role in determining aperture number and placement. Through the analysis of plants with abnormal number of apertures and of mutants with abnormal cytokinesis, we presented evidence that the tetrahedral microspore arrangement is not absolutely necessary (*MiMe* mutants), nor sufficient (*lsq* plants), for the development of three apertures. Similarly, positions of last-contact points arising between sister microspores appear not to serve as determinants for aperture number, since three or more apertures can arise in the microspores that have one (*osd1*, *tam-2*, *MiMe*), three (*lsq*), or zero (*tes*) contact points with their sisters and since apertures can develop in the absence of contact with the intersporal callose wall.

In addition to the last-contact points, it has been recently suggested that aperture positions might depend on patterns of callose deposition and may be determined by sites where callose is deposited last (in some cases called ‘additional callose deposits’), which may or may not coincide with the positions of the last-contact points [47,48]. In some species, most notably in *Typha latifolia* [48], such last-callose deposits are clearly visible since they do not lie at the intersporal callose walls, and indeed appear to exhibit strong correlation with positions of apertures. In our observations of tetrads and dyads, we therefore paid close attention to possible areas of additional callose deposition that could correlate with positions of ectopic apertures, yet so far in the Arabidopsis lines used here we did not observe any obvious additional callose deposits.

In contrast, we found that the factor that most strongly positively correlates with changes in number and positions of apertures is pollen ploidy, with higher ploidy typically associated with higher aperture number: e.g. while haploid Arabidopsis pollen usually develops three equidistantly placed equatorial apertures, independently of whether it is produced through a wild-type tetrad or a *MiMe* dyad, the diploid pollen mostly develops four or six apertures, and pollen with ploidy of 3n or higher develops more than six apertures that tend to coalesce into variable, ring-like patterns.

We note that changes in ploidy or ploidy-sensitive characteristics, like cell size, could also be an explanation for results of some previous studies, in which these characteristics have not been considered [22,49,50]. For example, abnormal aperture patterns were observed in the pollen derived from cells whose meiotic progress was disrupted by centrifugation or colchicine treatment [48,49]; also, a difference in aperture number was reported between the species of *Nicotiana sylvestris* (three apertures) and *N*. *tabacum* (four apertures) [22]. While models involving positioning of meiotic spindles, microtubular organizing centers, and radial arrays of microtubules were previously proposed to explain these data [15,22,49,50], ploidy is expected to be affected in all these cases, including Nicotiana species: *N*. *sylvestris* is diploid, whereas *N*. *tabacum* is its allotetraploid derivative [51].

Although ploidy-sensitive factors play a major role in determining aperture number and positions, additional ploidy-independent components might also be contributing to the aperture pattern specification. This conclusion is based on the analysis of the *osd1* and *tam-2* mutants, which primarily develop either four or six apertures and differ from *lsq* plants, which primarily have four apertures. Also, the pollen of *MiMe* mutants, despite being haploid, developed 6 apertures in about a quarter of cases, again pointing to the presence of ploidy-independent factors. Together, ploidy-sensitive and ploidy-independent mechanisms function upstream of the aperture factor INP1 and direct it to specific sites at the microspore periphery. INP1, which normally localizes to the positions of three apertures in developing microspores, accumulates at ectopic sites in microspores of higher ploidy, defining positions of future ectopic apertures.

How can ploidy contribute to the formation of more than the normal complement of apertures? One possibility is that, due to the increase in gene copy number, higher ploidy leads to higher levels of aperture factors, which, unable to fit into three ‘aperture-length-worth’ sites, are forced to form ectopic aperture sites. We demonstrated here that in the case of *INP1*, the only gene currently known to be directly involved in aperture formation, this does not seem to be the causative mechanism: in tetraploid plants with decreased expression of *INP1* in which apertures are shorter (and thus the ‘aperture-length’ potential is not reached), the number of apertures is the same as in tetraploid plants with normal *INP1*. Also, the higher levels of INP1 in the *DMC1pr*:*INP1-YFP* line do not result in increased aperture number. Taken together, this indicates that sites for aperture formation are specified before INP1 is brought to them. It remains a possibility that in higher-ploidy plants the increased levels of other, unknown, gene products cause these proteins to localize to more sites than normal and to attract or bring INP1 along.

An alternative possibility is that changes in microspore size, rather than changes in ploidy *per se*, are responsible for altered aperture number. Changes in volume and surface area likely affect mechanical properties of the microspore surface, such as curvature or surface tension of the plasma membrane or its attachment to and interactions with the overlying callose wall. Such changes could also affect the shape of the microspores or have an effect on the microspore cytoskeleton or on distribution of intracellular structures that might be involved in delivering INP1 to its peripheral positions. Additionally, changes in cell size might change concentrations of aperture-promoting or aperture-inhibiting factors. To distinguish between the effects of cell size vs. increased expression of unknown factors, one would need pollen in which one of the parameters (ploidy or size) changes, while the other stays the same. These two parameters, however, proved to be very challenging to uncouple in pollen: although it was reported that the Arabidopsis mutant *rhl2-1* might have such characteristics [52], with our imaging tools we found that diploid pollen in this mutant was not significantly smaller than diploid pollen of other genotypes (S5 Fig). Similarly, when we tested several normal-ploidy mutants that had enlarged somatic cells (*atkin13a* [53], *ARL-OE* [54], and *35Spr*::*ANT* [55]) to see if their pollen size might also be increased, we found that their pollen size was normal (S5 Fig). Therefore, additional approaches will have to be developed to distinguish between these two scenarios.

## Materials and Methods

### Plant material and growth conditions

Arabidopsis plants of the following genotypes were used: Columbia (Col-0), *lsq3*, *lsq6*, *lsq7* [30], *inp1-1* [21], *osd1-2* [31], *osd1-3* [31], *tam-2* [32], *tes* (SALK_113909), *MiMe* [31], *cenh3-1; GFP-tailswap* (CS66982) [38], *INP1pr*:*INP1-YFP*; *inp1* [21]; *DMC1pr*:*INP1-YFP; inp1* (to be described in detail elsewhere); *INP1pr*:*INP1-myc; inp1* #28 (short-aperture line) [21], and *INP1pr*:*INP1-myc; inp1* #10 (medium-aperture line) [21]. Diploid *tam-2* and *osd1* single and double mutant plants were recovered as segregating progeny of the corresponding diploid heterozygous plants (seeds from heterozygotes were provided by Raphaël Mercier and Robert Martienssen) by genotyping with primers listed in the S1 Table. For Brassica species and tetraploid accessions of Arabidopsis, the following seed stocks available from the Arabidopsis Biological Resource Center (ABRC) were used: *B*. *oleracea* (CS29002), *B*. *rapa* (CS29001), *B*. *napus* (CS29007), 4n Col (CS3151), 4n L*er* (CS3900), Wa-1 (CS6885). Plants were grown at 22°C with the 16-hour light: 8-hour dark cycle in the growth chambers or in the greenhouse at the Biotechnology facility at OSU. Maize pollen from the diploid (#102042, Hollick lab collection) and tetraploid (N105B and a derivative of N104B –Maize Coop accession numbers, maizegdb.org) plants was provided by Jay Hollick (OSU).

### Generation of the haploid *MiMe* plants

Plants that were triple heterozygotes for *atrec8-3*, *osd1-3*, and *atspo11-1-3* (*MiMe* heterozygotes) were identified from the F_1_ progeny of the cross between *atrec8-3/+* and *osd1-3/+; atspo11-1-3/+* (F_1_ seeds from this cross were kindly provided by Raphaël Mercier) by genotyping with primers listed in the S1 Table. These triple heterozygous plants were then crossed as males with *cenh3-1; GFP-tailswap* homozygous plants that were used as haploid inducers [38]. *cenh3-1; GFP-tailswap* homozygotes were identified from the *cenh3-1/+; GFP-tailswap* stock (CS66982) by their characteristic curled rosette leaf morphology and confirmed by genotyping with a *cenh3-1* dCAPS marker (S1 Table) [38]. Haploid progeny of this cross were readily identified by their distinctive morphology (narrow rosette leaves and small flowers), as described by Ravi and Chan [38], and the triple *atrec8; atspo11-1; osd1* (haploid *MiMe*) mutants were identified among the haploids by genotyping (primers listed in S1 Table). Unlike the other haploid genotypes generated by this cross, which were sterile, the triple *MiMe* haploid plants were fertile and produced 1n pollen via mitosis-like division.

### Colchicine treatment

To create plants with higher ploidy, shoot apical meristems of young plants were treated with colchicine. A 20-μl drop of 0.5% colchicine; 0.2% Silwet was applied twice onto shoot apices before bolting, with a 3-day interval between applications. Conspicuous, larger-than-normal flowers (often found on thicker-than-normal stems) in colchicine-treated plants were allowed to self-pollinate and their seeds were harvested. These progeny were then analyzed for characteristic increase in the size of plant organs and of pollen and for stable inheritance of these traits.

### Microscopy

For confocal microscopy of mature pollen grains, pollen was placed into a 5-μl drop of auramine O solution (0.001%; diluted in water from the 0.1% stock prepared in 50 mM Tris-HCl), allowed to hydrate, covered with a #1.5 coverslip, and sealed with nail polish. Pollen then was visualized with a 100x oil-immersion objective (NA = 1.4) on Olympus Fluoview1000 or Nikon A1+ confocal microscopes using FITC excitation/emission settings and 3x confocal zoom. To count apertures, images from the front and back view of the pollen grains were taken. If some apertures were present on sides of a pollen grain not directly visible by focusing on the front and on the back, then z-stacks were taken (step size = 300 nm) and 3D images were reconstructed using NIS Elements software v.4.20 (Nikon) and used for aperture counting. To determine the size of pollen grains in plants of different ploidy and genotypes, we used the surface areas of “front-view” pollen images (the surface closest to the microscope objective) as a proxy for cell size and measured these areas using NIS Elements and ImageJ.

For imaging tetrads and dyads, anthers were dissected out of stage-9 flower buds [56] and placed into the Vectashield anti-fade solution (Vector Labs, Burlingame, CA) supplemented with membrane stain CellMask Deep Red (Molecular Probes, Eugene, OR) (5 μg/ml) and calcofluor white (0.02%). Tetrads were released by covering anthers with a coverslip and applying gentle pressure on a coverslip. For imaging apertures at the tetrad and dyad stage, Vectashield was supplemented with DAPI (1 μg/ml). Tetrads and dyads were imaged on confocal Nikon A1+ with 100x oil-immersion objective (NA = 1.4) and 5x confocal zoom. YFP was excited with 514-nm laser and emission was collected at 522–555 nm, DAPI and calcofluor white were excited with 405-nm laser and collected at 424–475 nm, and CellMask Deep Red dye was excited with 640-nm laser and collected at 663–738 nm. Z-stacks of tetrads were obtained with a step size of 300 or 500 nm and 3D-reconstructed using NIS Elements v.4.20 (Nikon) or IMARIS (Bitplane) software.

To measure levels of YFP fluorescence, the *INP1pr*:*INP1-YFP* and *DMC1pr*:*INP1-YFP* tetrads were prepared simultaneously and imaged on the same day under identical acquisition conditions on confocal Nikon A1+. The mean YFP signal intensity of diffuse cytoplasmic INP1-YFP fluorescence was determined with the help of NIS Elements v.4.20 (Nikon) for one microspore per tetrad (n≥15 tetrads), using a single optical section with diffused fluorescence for each microspore. The optical sections containing assembled puncta of INP1-YFP at the periphery of microspores were excluded from the analysis.

To determine karyotypes of elongated cells in anther filaments [57], flowers were fixed in a 3:1 mixture of ethanol: acetic acid for 5 min and neutralized in 50 mM potassium phosphate buffer, pH 7.0. Anther filaments were dissected and placed in a drop of DAPI (10 μg/ml) in 80% glycerol. Confocal z-stacks of elongated cells were obtained and chromosomes counted using maximum intensity projections. Microspore mother cells undergoing meiosis were prepared and visualized as described [58].

### Quantitative RT-PCR

Total RNA was extracted from approximately 130 stage-9 *INP1pr*:*INP1-YFP; inp1-1* and *DMC1pr-INP1-YFP; inp1-1* buds (about 0.3–0.5 mm long) with TRIzol Reagent (Ambion), following the manufacturer’s instructions. RNA concentration was determined via spectrophotometry using Nanodrop (ND-1000; Thermo Scientific). To remove any contaminating genomic DNA, 5 μg of RNA was treated with 1.5 units of RQ1 RNase-Free DNAse (Promega) in 20 μl reactions at 37°C for 20 min, followed by addition of 0.8 μl of DNAse stop solution and DNAse inactivation at 75°C for 10 min. 8 μl of DNAse-treated RNA was pre-incubated with 1 μl of dNTPs (10 mM) and 1 μl of (dT)_20_ (50 μM) at 65°C for 5 min and then reverse-transcribed with the help of the SuperScript III Kit (Invitrogen) in a 20 μl reaction in the presence of 2 μl RT buffer (10x), 4 μl MgCl_2_ (25mM), 2 μl DTT (0.1M), 1 μl RNaseOUT (40 U/μl) and 1 μl SuperScript III reverse transcriptase (200 U/μl). The reactions were carried out at 50°C for 50 min, followed by incubation at 85°C for 5 min.

For qRT-PCR, the cDNA equivalents of 375 ng of initial RNA were amplified in 10-μl reactions using iQ SYBR Green Supermix (Bio-Rad) on a CFX96 real-time system (Bio-Rad). To amplify the wild-type transcripts produced from the *INP1-YFP* transgenes, the primers INP1-KF1 and INP1-LR [21] were used. To amplify the transcripts of the endogenous mutant *inp1-1* gene, the primers INP1-KF1mut and INP1-LR [21] were used. Primer specificity was confirmed previously [21]. qRT-PCR cycling conditions included an initial incubation at 95°C for 3 min, followed by 40 cycles of 95°C for 15 s and 60°C for 1 min. Four biological replicates and two technical replicates were used for both transgenic lines. After qRT-PCR, the amounts of both transcripts were calculated relative to standard curves obtained for serial dilutions of the wild-type and *inp1-1* genomic DNAs, respectively. For each sample, the amounts of the *INP1-YFP* transgenic transcripts were then normalized to the amounts of the endogenous *inp1-1* transcripts.

## Supporting Information

S1 Table Primers used for mutant and transgene genotyping. (PDF)

S1 Fig Pollen size exhibits strong correlation with pollen ploidy. Areas of pollen surface visible in the ‘front view’ images were measured for pollen of the genotypes examined in this study. Data are shown as mean ± SD. The pollen sizes are significantly different between the pollen grains of different ploidy (p-value < 0.05, indicated by *; the only exception is a comparison of *lsq3* and *tam-2-4n*, which are not statistically different (p-value = 0.07)). The pollen sizes are not different between pollen grains of different genotypes that have the same ploidy.
(PDF)

S2 Fig **Karyotypes of somatic elongated cells of anther filaments from (A) wild-type Columbia (2n) and (B) *lsq6*.** Chromosomes were stained with DAPI and visualized as brightly stained spots of centromeric heterochromatin using maximum intensity projections of confocal z-stacks. Scale bars = 5 μm.
(PDF)

S3 Fig Pollen from 3n plants is similar in size to pollen of wild-type 2n plants and usually has three apertures. (A, A’) Front and back of 1n wild-type pollen. (B, B’) Front and back of pollen from a 3n plant (F_1_ progeny of 2n x 4n cross). Scale bars = 10 μm.
(PDF)

S4 Fig Ring-like apertures that are common in 4n and 3n pollen may result from the initiation of multiple ectopic apertures that get connected with each other. (A) Incompletely closed ring in a 4n pollen grain from a 4n *osd1* plant. (B, B’) Front and back view of a 3n pollen grain with ring-like apertures from a 6n plant.
(PDF)

S5 Fig Pollen size exhibits strong correlation with pollen ploidy. Areas of pollen surface visible in the ‘front view’ images were measured for pollen grains of several lines (boxed graphs) reported to have normal ploidy but increased size of somatic cells *(ARL-OE*, *atkin13a*, *35S*::*ANT*), as well as for a tetraploid line (*rhl2-1-4n*) that was reported to have smaller pollen grains than other tetraploid lines. In all cases, the pollen sizes were not significantly different from pollen sizes of control lines with the same ploidy. Data are shown as mean ± SD.
(PDF)

S1 Movie Volume reconstruction of a z-stack of confocal sections through a wild-type tetrad of microspores. Microspores express INP1-YFP (yellow). Microspore surface was transiently labeled with DAPI (blue).
(MOV)

S2 Movie Volume reconstruction of a z-stack of confocal sections through a *lsq6* tetrad of microspores. Microspore surface was transiently labeled with DAPI (green) and plasma membrane was stained with CellMask Deep Red (magenta) to highlight positions of developing apertures.
(MOV)
