# Supplementary material for: A Ploidy-Sensitive Mechanism Regulates Aperture Formation on the Arabidopsis Pollen Surface and Guides Localization of the Aperture Factor INP1
Source: PLoS Genet. 2016 May 13;12(5):e1006060. doi: 10.1371/journal.pgen.1006060 (PMC4866766; doi:10.1371/journal.pgen.1006060)
Supplement: S1 Table — (PDF) [file pgen.1006060.s001.pdf]

S1 Table. Primers used for mutant and transgene genotyping.

Mutant/Transgene	Primers for wild-type allele	Primers for mutant allele
osd1-2	osd1-2-GT21481U (CCGGTGTTCTTGTGACTCG); osd1-2-GT21481L (GCAGATTCCTAATTCAGCTC)	osd1-2-GT21481U; Ds3-4 (CCGTCCCGCAAGTTAAATATG)
osd1-3	osd1-3-Fw (CTCATTGTCTCTCTACACAGTTTTGGAGTT); osd1-3-Rev (TCGCATATGGTCTCTTCTCCTAAGTAAAGA)	T-DNA-Fw (CTGGGAATGGCGAAATCAAGGCATC); osd1-3-Rev
tam-2	tam-2-N874380U (GACTTGATGGATCCACAGC); tam-2-N874380L (CAGAAATCCTCCACTTGCG)	LB3Sail (TAGCATCTGAATTTCATAACCAATCTCGATACAC); tam-2-N874380L
spo11-1-3	Atspo11-1-3-N646172U (AATCGGTGAGTCAGGTTTCAG); Atspo11-1-3-N646172L (CCATGGATGAAAGCGATTTAG)	LBSalk2 (GCTTTCTTCCCTTCCTTTCTC); Atspo11-1-3-N646172L
rec8-3	Atrec8-3-N836037U (CTCATATTCACGGTGCTCCC); Atrec8-3-N836037L (GGGGGAAAAGAGAAAGGTTC)	LB3Sail; Atrec8-3-N836037L
cenh3-1	cenh3-1-F (GGTGCGATTTCTCCAGCAGTAAAAATC); cenh3-1-R (CTGAGAAGATGAAGCACCGGCGATAT); digest with *EcoR*V - fragment is cut	cenh3-1-F; cenh3-1-R; digest with *EcoR*V – fragment remains uncut
inp1-1	22600-DF (CCATTTAGACAAGGGCTTG); 22600-DR (AACTTGATACGACGAGACC); digest with *Sac*I – fragment remains uncut	22600-DF; 22600-DR; digest with *Sac*I – fragment is cut
INP1pr:INP1-YFP	22600-BF (ACGACGAAGACGAGGAAAC) EYFP-3 (GTTCACCTTGATGCCGTTCTTCTG)	
