# Supplementary material for: A Ploidy-Sensitive Mechanism Regulates Aperture Formation on the Arabidopsis Pollen Surface and Guides Localization of the Aperture Factor INP1
Source: PLoS Genet. 2016 May 13;12(5):e1006060. doi: 10.1371/journal.pgen.1006060 (PMC4866766; doi:10.1371/journal.pgen.1006060)
Supplement: S1 Fig — Areas of pollen surface visible in the ‘front view’ images were measured for pollen of the genotypes examined in this study. Data are shown as mean ± SD. The pollen sizes are significantly different between the pollen grains of different ploidy (p-value < 0.05, indicated by *; the only exception is a comparison of lsq3 and tam-2-4n, which are not statistically different (p-value = 0.07)). The pollen sizes are not different between pollen grains of different genotypes that have the same ploidy. (PDF) [file pgen.1006060.s002.pdf]

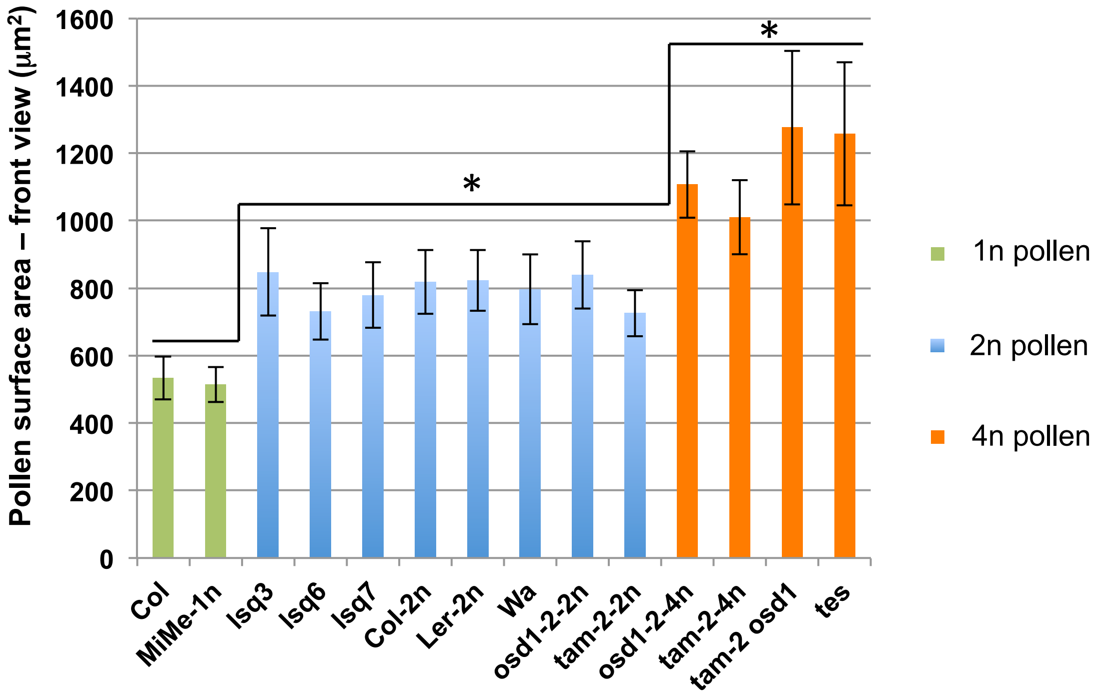

Supplemental Figure 1. Pollen size exhibits strong correlation with pollen ploidy. Areas of pollen surface visible in the 'front view' images were measured for pollen of the genotypes examined in this study. Data are shown as mean $\pm$ SD. The pollen sizes are significantly different between the pollen grains of different ploidy (p-value < 0.05, indicated by *; the only exception is a comparison of *lsq3* and *tam-2-4n*, which are not statistically different (p-value=0.07)). The pollen sizes are not different between pollen grains of different genotypes that have the same ploidy.
