# Supplementary material for: A Ploidy-Sensitive Mechanism Regulates Aperture Formation on the Arabidopsis Pollen Surface and Guides Localization of the Aperture Factor INP1
Source: PLoS Genet. 2016 May 13;12(5):e1006060. doi: 10.1371/journal.pgen.1006060 (PMC4866766; doi:10.1371/journal.pgen.1006060)
Supplement: S2 Fig — Karyotypes of somatic elongated cells of anther filaments from (A) wild-type Columbia (2n) and (B) lsq6. Chromosomes were stained with DAPI and visualized as brightly stained spots of centromeric heterochromatin using maximum intensity projections of confocal z-stacks. Scale bars = 5 μm. (PDF) [file pgen.1006060.s003.pdf]

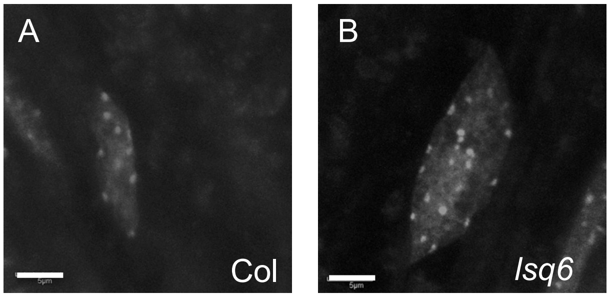

Supplemental Figure 2. Karyotypes of somatic elongated cells of anther filaments from (A) wild-type Columbia (2n) and (B) *lsq6*. Chromosomes were stained with DAPI and visualized as brightly stained spots of centromeric heterochromatin using maximum intensity projections of confocal z-stacks. Scale bars = 5 μm.
