# Supplementary material for: A Ploidy-Sensitive Mechanism Regulates Aperture Formation on the Arabidopsis Pollen Surface and Guides Localization of the Aperture Factor INP1
Source: PLoS Genet. 2016 May 13;12(5):e1006060. doi: 10.1371/journal.pgen.1006060 (PMC4866766; doi:10.1371/journal.pgen.1006060)
Supplement: S3 Fig — (A, A’) Front and back of 1n wild-type pollen. (B, B’) Front and back of pollen from a 3n plant (F1 progeny of 2n x 4n cross). Scale bars = 10 μm. (PDF) [file pgen.1006060.s004.pdf]

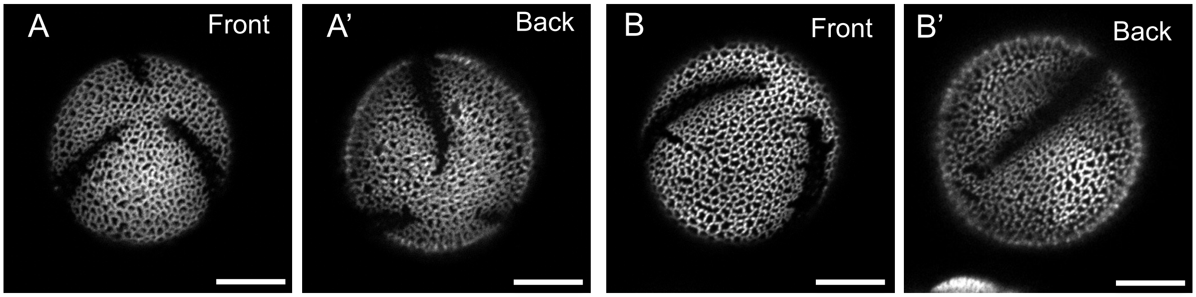

Supplemental Figure 3. Pollen from 3n plants is similar in size to pollen of wild-type 2n plants and usually has three apertures. (A, A') Front and back of 1n wild-type pollen. (B, B') Front and back of pollen from a 3n plant (F_1 progeny of 2n x 4n cross). Scale bars = 10 μm.
