# Supplementary material for: A Ploidy-Sensitive Mechanism Regulates Aperture Formation on the Arabidopsis Pollen Surface and Guides Localization of the Aperture Factor INP1
Source: PLoS Genet. 2016 May 13;12(5):e1006060. doi: 10.1371/journal.pgen.1006060 (PMC4866766; doi:10.1371/journal.pgen.1006060)
Supplement: S4 Fig — (A) Incompletely closed ring in a 4n pollen grain from a 4n osd1 plant. (B, B’) Front and back view of a 3n pollen grain with ring-like apertures from a 6n plant. (PDF) [file pgen.1006060.s005.pdf]

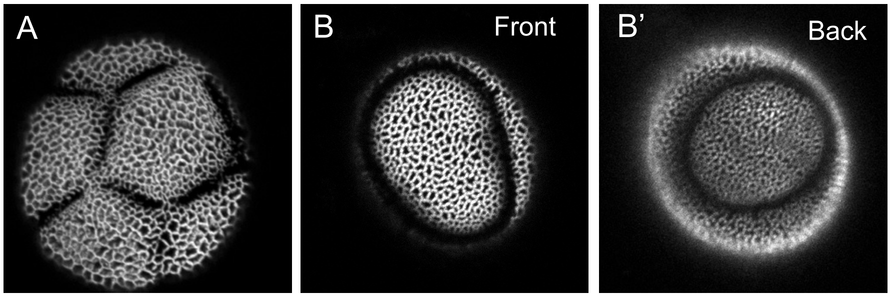

Supplemental Figure 4. Ring-like apertures that are common in 4n and 3n pollen may result from the initiation of multiple ectopic apertures that get connected with each other.(A) Incompletely closed ring in a 4n pollen grain from a 4n *osd1* plant. (B, B’) Front and back view of a 3n pollen grain with ring-like apertures from a 6n plant.
