# Supplementary material for: A Ploidy-Sensitive Mechanism Regulates Aperture Formation on the Arabidopsis Pollen Surface and Guides Localization of the Aperture Factor INP1
Source: PLoS Genet. 2016 May 13;12(5):e1006060. doi: 10.1371/journal.pgen.1006060 (PMC4866766; doi:10.1371/journal.pgen.1006060)
Supplement: S5 Fig — Areas of pollen surface visible in the ‘front view’ images were measured for pollen grains of several lines (boxed graphs) reported to have normal ploidy but increased size of somatic cells (ARL-OE, atkin13a, 35S::ANT), as well as for a tetraploid line (rhl2-1-4n) that was reported to have smaller pollen grains than other tetraploid lines. In all cases, the pollen sizes were not significantly different from pollen sizes of control lines with the same ploidy. Data are shown as mean ± SD. (PDF) [file pgen.1006060.s006.pdf]

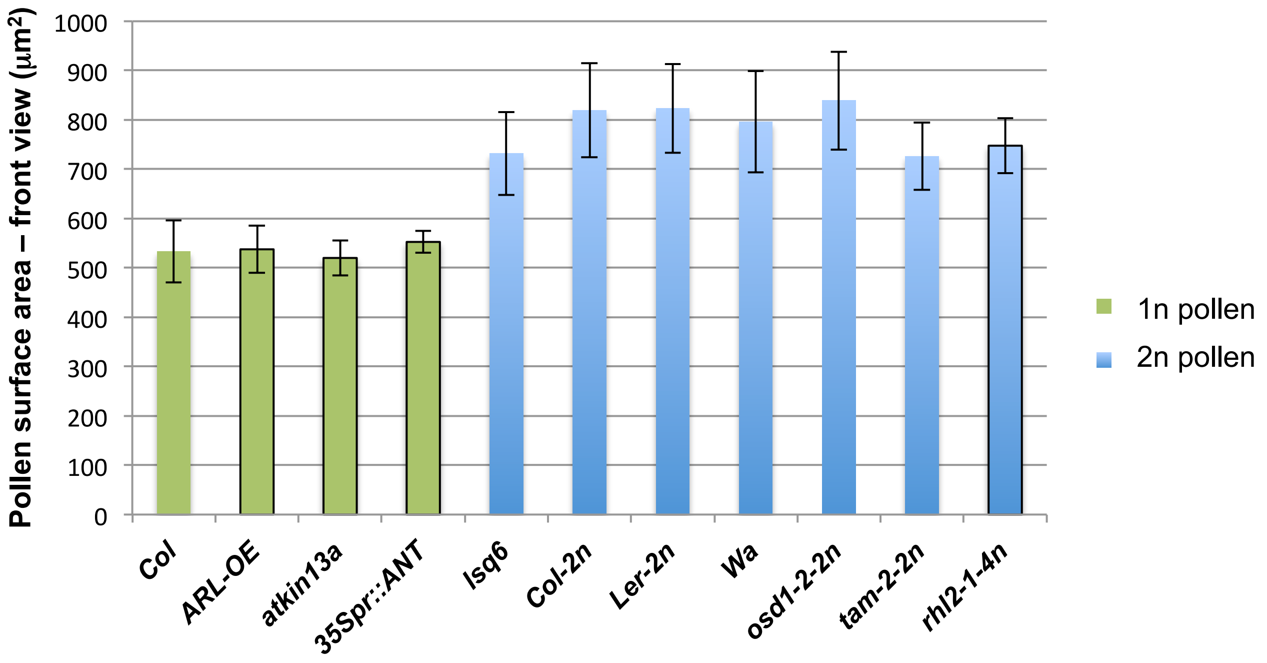

Supplemental Figure 5. Pollen size exhibits strong correlation with pollen ploidy. Areas of pollen surface visible in the 'front view' images were measured for pollen grains of several lines (boxed graphs) reported to have normal ploidy but increased size of somatic cells *(ARL-OE*, *atkin13a*, *35S::ANT*), as well as for a tetraploid line (*rhl2-1-4n*) that was reported to have smaller pollen grains than other tetraploid lines. In all cases, the pollen sizes were not significantly different from pollen sizes of control lines with the same ploidy. Data are shown as mean ± SD.
